# ZASC1 knockout mice exhibit an early bone marrow-specific defect in murine leukemia virus replication

**DOI:** 10.1186/1743-422X-10-130

**Published:** 2013-04-24

**Authors:** Shannon Seidel, James Bruce, Mathias Leblanc, Kuo-Fen Lee, Hung Fan, Paul Ahlquist, John AT Young

**Affiliations:** 1 Nomis Center for Immunobiology and Microbial Pathogenesis, The Salk Institute for Biological Studies, La Jolla, CA, USA; 2 Division of Biology, University of California San Diego, San Diego, CA, USA; 3 Institute for Molecular Virology, University of Wisconsin, Madison, WI, USA; 4 Morgridge Institute for Research, Madison, WI, USA; 5 The Salk Institute for Biological Studies, La Jolla, CA, USA; 6 Clayton Foundation Laboratories for Peptide Biology, The Salk Institute for Biological Studies, La Jolla, CA, USA; 7 Department of Molecular Biology and Biochemistry, University of California Irvine, Irvine, CA, USA; 8 Howard Hughes Medical Institute, University of Wisconsin, Madison, WI, USA

**Keywords:** ZASC1, Retrovirus, Murine leukemia virus, Host cell factors, Mouse model

## Abstract

**Background:**

ZASC1 is a zinc finger-containing transcription factor that was previously shown to bind to specific DNA binding sites in the Moloney murine leukemia virus (Mo-MuLV) promoter and is required for efficient viral mRNA transcription (J. Virol. 84:7473-7483, 2010).

**Methods:**

To determine whether this cellular factor influences Mo-MuLV replication and viral disease pathogenesis *in vivo*, we generated a ZASC1 knockout mouse model and completed both early infection and long term disease pathogenesis studies.

**Results:**

Mice lacking ZASC1 were born at the expected Mendelian ratio and showed no obvious physical or behavioral defects. Analysis of bone marrow samples revealed a specific increase in a common myeloid progenitor cell population in ZASC1-deficient mice, a result that is of considerable interest because osteoclasts derived from the myeloid lineage are among the first bone marrow cells infected by Mo-MuLV (J. Virol. 73: 1617-1623, 1999). Indeed, Mo-MuLV infection of neonatal mice revealed that ZASC1 is required for efficient early virus replication in the bone marrow, but not in the thymus or spleen. However, the absence of ZASC1 did not influence the timing of subsequent tumor progression or the types of tumors resulting from virus infection.

**Conclusions:**

These studies have revealed that ZASC1 is important for myeloid cell differentiation in the bone marrow compartment and that this cellular factor is required for efficient Mo-MuLV replication in this tissue at an early time point post-infection.

## Background

Transcription of retroviral genomes involves numerous cellular transcription factors that bind to the unique 3′ (U3) enhancer element located in the viral promoter [1-4]. These transcription factors can dictate viral cell-type tropism as well as the types of viral diseases that are elicited [5-7]. For example, in the case of Moloney murine leukemia virus (Mo-MuLV) mutations in the core binding site primarily induce erythroleukemia instead of thymic lymphoma [7].

Previously, our groups employed a forward genetic screen that identified ZASC1 (Zinc Finger Amplified in Esophageal Squamous Carcinoma 1 or ZNF639) as a cellular transcription factor that binds three specific DNA sequences located within the U3 region of the Mo-MuLV genome [8]. ZASC1 is a 9-C_2_H_2_ zinc finger protein [9] that binds and translocates alpha-N-catenin to the nucleus [10] and also interacts with the CREB binding protein (CBP) [11]. We also showed that ZASC1 promotes viral gene expression in established cultured cell lines [8]. More recently we have found that ZASC1 is also required for efficient HIV-1 gene expression by a mechanism that is associated with recruitment of a viral Tat/cellular pTEFb complex to the viral core promoter (Bruce *et al.* in preparation).

To determine the role of ZASC1 in retroviral replication and disease pathogenesis *in vivo*, we have now generated a ZASC1^−/−^ mouse strain. We report here that ZASC1-knockout mice exhibit an early viral replication defect in bone marrow that is associated with a specific defect in myeloid cell differentiation.

## Results

### Generation and characterization of ZASC1^−/−^ mice

**Figure 1:**
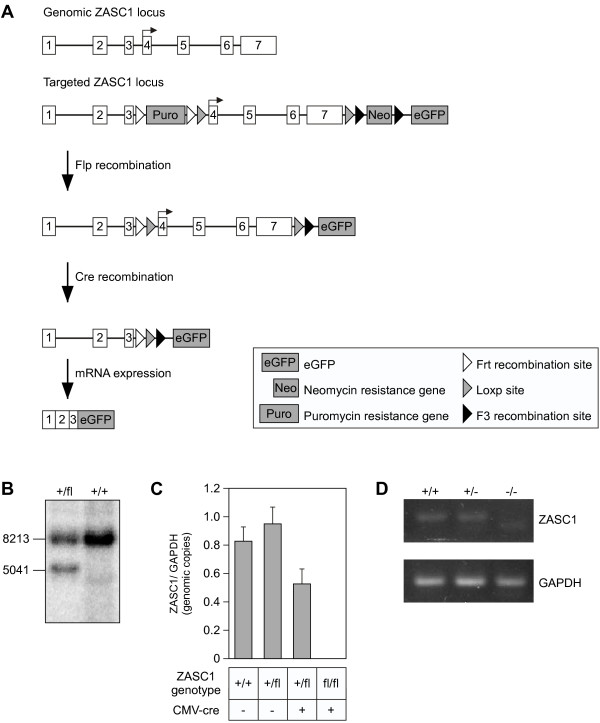
**Generating the ZASC1**^**−/−**^**mouse model.A**.) Schematic representation of the ZASC1 genomic locus (top) and ZASC1 locus after inserting loxp sites via cassettes containing puromycin and neomycin resistance genes. White boxes represent exons. Black line represents intronic or other sequence. Black arrow indicates translational start site. eGFP with a splice acceptor sequence was inserted downstream of the Neomycin targeting in an effort to express eGFP from recombined ZASC1 gene. **B**.) Southern blot of Sph1-digested DNA showing homologous recombination at the ZASC1 locus in genomic DNA isolated from 129 J ES cells; the 8,213 bp band represents the ZASC1 genomic locus and the 5,041 bp band represents the floxed ZASC1 locus. **C**.) ZASC1 gene copy number measured by qPCR, normalized to GAPDH gene copy number, in animals with different ZASC1 genotypes. **D**.) ZASC1 RNA expression in whole blood cells from each ZASC1 genotype measured by RTPCR. Lower band in ZASC1^−/−^ lane represents primer-dimer.

To address the potential role of ZASC1 in wild type (WT) Mo-MuLV replication and disease pathogenesis *in vivo*, we generated a *ZASC1* conditional knockout mouse (Figure 1A). *ZASC1* contains 7 exons and the open reading frame is located in exons 4–7. LoxP sites were introduced into the DNA sequences flanking *ZASC1* exons 4–7 so that the complete *ZASC1* open reading frame was deleted following Cre-mediated recombination. The first loxP site was inserted into the intron between exons 3 and 4 using a puromycin (puro) selection cassette. A second loxP site was inserted downstream of exon 7 and the polyadenylation signal as part of a neomycin (neo) cassette to select for insertion of this site. The neo and puro cassettes, flanked by F3 and Frt sites respectively, were excised in the presence of Flp recombinase.

The targeting vector was inserted into 129/J ES cells and 294 G418-resistant colonies were selected. Embryonic stem (ES) cell clones were screened for homologous recombination by southern blot analysis detecting a 5041 bp restriction fragment that was diagnostic of homologous recombination (Figure 1B). Positive cell clones were subsequently screened to identify those that also contained the upstream arm of the targeting vector, and the two clones with the best morphology were chosen for injection into C57bl/6 blastocysts. Chimeric males were bred for germline transmission of the *ZASC1* allele and screened by coat color.

After germline transmission was achieved, mice were crossed to ß-actin-Flp^+^ C57bl/6 in order to remove the antibiotic resistance cassettes in the targeting construct to generate ZASC1^+/fl^ mice. Finally, ZASC1^+/fl^ mice were bred to a CMV-Cre C57bl/6 to generate ZASC1^−/−^ mice. Future generations of ZASC1^+/−^ heterozygotes were bred to generate ZASC1^−/−^ offspring. Quantitative PCR analysis of tail genomic DNA confirmed the complete deletion of exons 4–7 in ZASC1 from the ZASC1^fl/fl^ animals expressing CMV-cre (Figure 1C). RT-PCR analysis of whole blood samples demonstrated that ZASC1 expression was also completely absent in ZASC1^−/−^ mice (Figure 1D). ZASC1^−/−^ mice were born at normal Mendelian ratios and had similar weights and lifespans as compared to their WT littermates (data not shown). Furthermore, deletion of ZASC1 did not cause any obvious physical or behavioral defects (data not shown).

### ZASC1^−/−^ mice have normal levels of B and T lymphocytes

**Figure 2:**
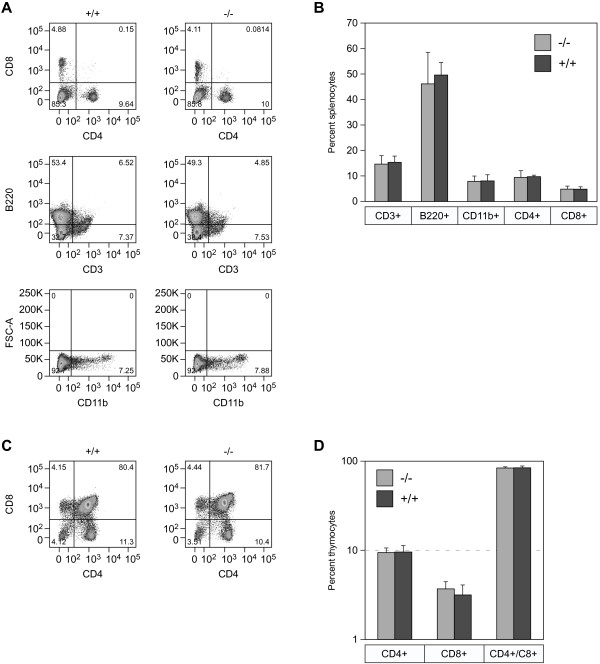
**ZASC1 is not required for normal differentiation of splenocytes or thymocytes.** Spleen and thymus from ZASC1^+/+^ and ZASC1^−/−^ animals were harvested and stained using α-CD3, α-B220, α-CD11b, α-CD4, and α-CD8 fluorophore-conjugated antibodies and analyzed by flow cytometry. **A**.) Example of gating for mature cell populations after FSC/SSC analysis to gate on live cells. Top panel shows T-cell subsets (CD4+ and CD8+). Middle panel shows B-cells (B220) and T-cells (CD3). Bottom panel shows monocytes/macrophages (CD11b). **B**.) Graphical representation of the FACS plots shown in panel **A**. **C**.) Example of gating for CD4+, CD8+, and double positive thymocytes. **D**.) Graphical representation of thymocyte populations shown in panel **C**. N=3 mice per group with littermate controls. Error bars = SD. No statistical significance was observed between the different animal genotypes by two-tailed T-test.

Because ZASC1 is expressed ubiquitously and MLV primarily infects hematopoietic cell types, we first analyzed the major hematopoietic cell populations in lymphoid organs by flow cytometry. These studies revealed no deficiency in the proportion of T-cells or B-cells in the spleen (Figure 2A and 2B) and thymus (Figure 2C and 2D) of ZASC1^−/−^ mice as compared to WT mice. Moreover, T-cells derived from young ZASC1^−/−^ mice showed no functional defect when tested for activation *in vitro* (Additional file 1: Figure S1). Therefore, we conclude that ZASC1 deficiency does not significantly alter mouse splenic or thymic B or T cell populations.

### ZASC1-deficient mice exhibit an altered bone marrow common myeloid progenitor cell population

**Figure 3:**
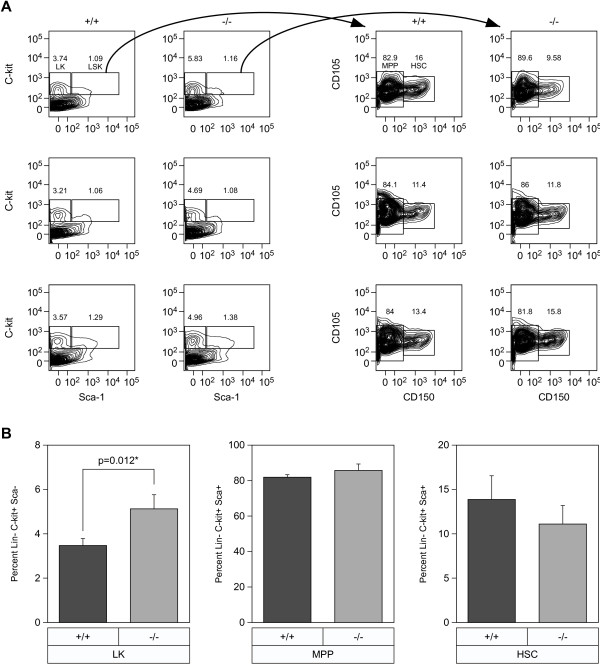
**ZASC1**^**−/−**^**mice have increased LK cell compartments in the bone marrow. A**.) FACS plots gating hematopoietic progenitor populations in the bone marrow. Antibodies used: α-c-kit, α-sca-1, α-CD105, α-CD150, and lineage markers (α-CD11b, α-Gr-1, α-Ter119, α-CD3, α-Ly6C, α-CD19, α-CD11c). LK (Lin^-^ Sca-1^-^ C-kit^+^), LSK (Lin^-^ Sca-1^+^ C-kit^+^), MPP (multipotent progenitor). and HSC (hematopoietic stem cell) populations within the bone marrow were gated as labeled in the top left box. **B**.) Graphical representation of the LK, LSK and HSC populations from multiple litters. N=3 mice per group. Mean average values. Error bars indicate SD. Two-tailed T-test was used to measure statistically significant differences between ZASC1^+/+^ and ZASC1^−/−^ populations. Significant p-value is indicated.

Previously, myeloid cell populations in the bone marrow were shown to be among the first cells infected by Mo-MuLV [12]. Therefore, flow cytometric analysis was used to examine the progenitor cell populations in the bone marrow. These cells were identified by a lack of lineage specific markers (lin-) and expression of sca-1 and c-kit on their surface. The lin-sca+kit+ (LSK) population contained the earliest bone marrow progenitors: hematopoietic stem cells (HSCs) were identified by gating on the CD105+ and CD150+ population whereas multipotent progenitors (MPPs) were identified by gating on the CD105+ CD150- cell subset, as described in [13]. The LK compartment (lin-sca-kit+) is known to contain myeloid and erythroid progenitors (Additional file 2: Figure S2) [14]. We found that HSC and MPP populations were comparable between ZASC1^−/−^ and ZASC1^+/+^ mice, but there was a statistically significant 1.5-fold increase (p-value = 0.012) in the heterogenous lin-ska-kit+ (LK) compartment in all ZASC1-deficient animals tested (Figure 3A and 3B). This compartment contains common myeloid progenitor cells and downstream myeloid precursors with no long-term repopulation potential [14]. We conclude that ZASC1 deficiency specifically leads to altered common myeloid progenitor cell differentiation in the bone marrow.

### Early defect in Mo-MuLV replication in the bone marrow of ZASC1^−/−^ mice

To address the role of ZASC1 in early retrovirus infection *in vivo,* a preliminary time course of infection was performed in mixed background 129 J and C57bl/6 mice with ZASC1^+/+^, ZASC1^+/−^ and ZASC1^−/−^ genotypes. Neonatal mice were inoculated p2-p3 by i.p. injection and viral titers were measured in the spleen, thymus, and bone marrow using an established focal immunofluorescence assay [15] at various time points post infection (7, 10, or 13 days) (Additional file 3: Figure S3). The virus titers in the bone marrow of ZASC1^−/−^ mice were significantly lower than those found in ZASC1^+/−^ mice 10 days post infection (p-value = 0.03).

**Figure 4:**
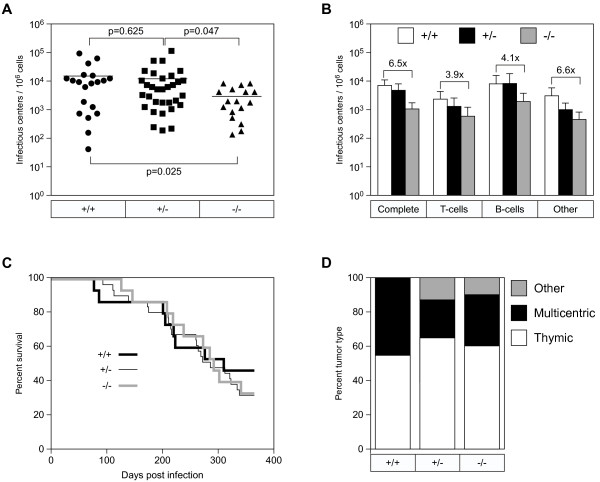
**Mo-MuLV infection is delayed in ZASC1**^**−/−**^**mice at an early time point. A**.) The offspring of ZASC1^+/−^ breeding pairs were inoculated i.p. with Mo-MuLV at p2-p3. Bone marrow from infected animals was harvested 10 days post infection along with tails to determine ZASC1 genotype. Virus infection was measured by focal immunofluorescence assay to determine infectious centers per 10^6^ cells. Each point represents a single animal: ZASC1^+/+^ (circles), ZASC1^+/−^ (squares), and ZASC1^−/−^ (triangles). Mean average values indicated by horizontal bar. Statistically significance measured by two-tailed T-test. p-values indicated on the graph. **B**.) Infections were completed as in panel **A**, but bone marrow was pooled by genotype before magnetic cell sorting was used to purify T-cells and B-cells from the bone marrow. “Complete” is unsorted bone marrow. “T-cells” were isolated from bone marrow using α-CD90.2 conjugated Dynabeads. “B-cells” were isolated from bone marrow using α-B220 conjugated Dynabeads. Focal immunofluorescence assay was used to quantify infected cells in each population. Graph is mean average levels of infection in WT cells. N=3 litters of mice pooled by genotype. Error bars show SD. Statistical significance measured using one-way ANOVA. **C**.) Kaplan-Meier plot comparing survival of Mo-MuLV-infected animals of ZASC1^+/+^ (thick black), ZASC1^+/−^ (thin black) and ZASC1^−/−^ (thick gray) genotypes. Litters of mice from ZASC1+/− breeding pairs were infected p2-p3 and were allowed to develop disease. Animals that had not exhibited overt signs of disease were terminated 1 year post-infection. ZASC1^+/+^ (N=15), ZASC1^+/−^ (N=31), ZASC1^−/−^ (N=15). **D**.) Stacked graph of tumor types by ZASC1 genotype.

To investigate this early defect in Mo-MuLV replication in the bone marrow in more detail, the study sample size was increased and bone marrow samples were harvested at 10 days post-infection. This analysis revealed a highly statistically significant 5.1-fold (p-value = 0.025) and 4.2-fold (p-value = 0.047) decrease in MLV replication in ZASC1^−/−^ mice as compared to ZASC1^+/+^ or ZASC1^+/−^ mice respectively (Figure 4A).

To determine if this replication defect was specific for one or more cell types in the bone marrow, we measured the virus titers in T-cell, B-cell, and in non-B/T cells in samples taken from several animals that were pooled by genotype. Mature T-cells and B-cells were immunopurified using CD90.2- or B220-coated magnetic beads respectively and flow cytometry was used to confirm cell population purity and the efficiency of T/B cell depletion (data not shown). Consistent with our previous results, the complete bone marrow samples derived from ZASC1^−/−^ animals supported 6.5-fold less Mo-MuLV replication as compared to ZASC1^+/+^ (Figure 4B). Strikingly, we found similar differences in virus titer when we examined the bone marrow-derived T-cell, B-cell, and non-B/T cell populations (Figure 4B). Therefore, the difference in virus infection seen between ZASC1^−/−^ versus ZASC1^+/+^ animals at an early time point appears to apply generally to all cell types in the bone marrow.

We also asked whether ZASC1 plays a role in long-term Mo-MuLV pathogenesis by infecting neonatal mice with Mo-MuLV at the p2-p3 stage and monitoring for up to 1 year for tumorigenesis. Moribund animals were analyzed for tumor type, timing, and severity. Despite the early virus replication defect in the bone marrow, we found that ZASC1 had no significant impact on disease outcome (Figure 4C and 4D).

## Discussion

In this study, we developed a gene knockout model to investigate the roles of *ZASC1* in murine development and retroviral pathogenesis. This cellular gene was previously shown to be important for efficient Mo-MuLV infection in established cultured cell lines [8]. ZASC1-deficient mice developed normally, had no fertility defects, and exhibited normal T and B lymphocyte numbers as well as normal levels of bone marrow hematopoietic stem cells and multipotential progenitor cells. However, ZASC1-deficiency was associated with a significant increase in a myeloid progenitor cell population (heterogenous lin-ska-kit+ or LK cells). The LK cell compartment includes the common myeloid progenitor (CMP), which ultimately gives rise to megakaryocytes, erythrocytes, monocytes, granulocytes, and osteoclasts. We also observed a specific defect in Mo-MuLV infection in the bone marrow at an early time point post-infection. This defect was seen with both lymphocyte and non-lymphocyte populations. Despite this early block to bone marrow infection, long-term Mo-MuLV-induced disease was unaffected by this cellular factor.

Previous studies had shown that early infection of the bone marrow correlates with leukemogenicity of Mo-MuLV [16]. Furthermore, cells of the myeloid lineage make up the majority of cell types infected during early stage Mo-MuLV infection after i.p. inoculation [12]. Specifically, osteoclasts and osteoclast precursors are among the first cells infected and these cell types are derived from the myeloid lineage [17]. Consequently, we envision at least two distinct models to explain the effect of ZASC1-deficiency on early Mo-MuLV replication in the bone marrow. The first model predicts that the defect seen with ZASC1^−/−^ bone marrow myeloid cell differentiation is responsible for the reduced level of initial infection. Reduced infection of these cells could then translate into a lower level of initial virus spread to other cell types in the bone marrow, including lymphocytes. These initial effects would presumably be subsequently masked once the virus has been amplified in the other bone marrow cell populations, explaining why we observed no differences in virus titer in this compartment at later time points or in long-term disease pathogenesis.

An alternative model is that ZASC1-deficiency leads to a more general block to virus replication (presumably at the level of viral gene expression) in all of the target cells contained in the bone marrow. However, for this model to be correct such an effect would have to be bone marrow-specific because ZASC1-deficiency was not associated with low virus titers in samples taken early post-infection from either splenic or thymic T- or B-cell populations. Future studies will be aimed at discriminating between these two different models and exploring the putative role of ZASC1 in myeloid cell differentiation.

## Conclusions

We have generated a ZASC1 knockout mouse and demonstrated that this mouse model has no obvious developmental or behavioral defects. When challenged with Mo-MuLV infection, ZASC1 deficient animals exhibited an early defect in Mo-MuLV replication in the bone marrow compartment. Further analysis of this compartment revealed an increase in the LK cell population, a group of progenitor cells that give rise to myeloid cell types including osteoclasts. Since osteoclasts in the bone marrow are among the first cell types infected by Mo-MuLV, these data suggest that ZASC1 might influence the number of these target cells available for establishing Mo-MuLV infection. Alternatively, ZASC1 might directly influence Mo-MuLV transcription in the bone marrow niche. Further studies will be needed to distinguish between these two possible models.

## Methods

### Generation of the ZASC1 knockout mice

A ZASC1 targeting vector was cloned using a recombineering approach, described in [18]. Briefly, loxP sites were introduced in the intron between *ZASC1* exons 3 and 4 and downstream of exon 7 with puromycin and neomycin resistance cassettes. The integrity of the ZASC1 targeting vector was confirmed by PCR amplification and DNA sequencing of cassette junctions as well as by restriction enzyme digestion. 129/J5 embryonic stem (ES) cells were electroporated (BioRad GenePulser) with the ZASC1 targeting vector and selected for integration in culture medium containing 200 μg/ml G418. G418 positive ES cell clones were analyzed for homologous recombination by Southern blot analysis at each homologous arm.

The Salk Institute Transgenic Mouse Core Facility performed blastocyst injections and highly chimeric males (by coat color) were bred with C57bl/6 females. Progeny with germline transmission of the targeted *ZASC1* locus were bred with a C57bl/6 transgenic mouse expressing FLP recombinase from the human Beta-actin promoter (Jackson Laboratory Stock Number 005703). Mice with FLP recombination were then bred with CMV-Cre mice (Jackson Laboratory Stock Number 006054).

### Genotyping

Tails collected from 14-day old pups were incubated overnight in tail lysis buffer (100 mM Tris pH 8, 5 mM EDTA, 2% SDS and 200 mM NaCl + Proteinase K) at 55°C for in house genotyping. DNA was purified by ethanol precipitation and dried pellets were resuspended in 500 μl TE. 1 μl diluted tail DNA samples were added to a 10 μl qPCR reaction mix that also included genotyping primers and FAST SYBR Green Master Mix (Applied Biosystems). Germline transmission of the ZASC1 knockout locus was detected with primer pair 5′-TCAGTGCATCAGCATACTCAAG-3′ and 5-CTGGTGGTTCTTCATCGGC-3′ and then normalized to GAPDH using primer pair 5′-ACCCAGAAGACTGTGGATGG-3′ and 5′-CACATTGGGGGTAGGAACAC-3′. qPCR analysis was performed using the Applied Biosystems ViiA 7 instrument. Genotyping for cre-recombinase, flp-recombinase and the floxed ZASC1 allele was done through a professional genotyping service (Transnetyx Inc.).

### Flow cytometric analysis

Spleen and thymus samples were harvested from euthanized 4-week old animals and ground into a single cell suspension through a 70 μM cell strainer and incubated in ACK buffer (Life Technologies) to lyse red blood cells. Bone marrow samples were harvested from euthanized 10-day old animals by crushing bones and straining through a 70 μM filter. Cells were resuspended in staining buffer (3% BSA in PBS) on ice and incubated with antibodies for 30 minutes before washing away unbound antibodies. For spleen analysis, fluorescently-labeled α-CD3, α-B220, α-CD11b, α-CD4, and α-CD8 antibodies were used (eBiosciences). For thymus analysis, fluorescently-labeled α-CD4 and α-CD8 antibodies were used (eBiosciences). For bone marrow, α-c-kit, α-sca-1, α-CD105, α-CD150, and lineage markers (α-CD11b, α-Gr-1, α-Ter119, α-CD3, α-Ly6C, α-CD19, α-CD11c) were used (eBiosciences and Steve Hedrick Lab, UCSD). Samples were analyzed using the FACSAria III (BD) instrument and FlowJo software (Tree Star, Inc.).

### Mo-MuLV production and titer determination

Virus was generated in extracellular supernatants from NIH 3T3-43D cells (Fan Lab, UC Irvine), filtered through a 0.45 micron filter, aliquotted and stored at −80°C. Viral titers were determined by Focal Immunofluorescence Assay (FIA) in the presence of polybrene as described previously [15]. Virus-infected cells were identified by staining with 538 antibody-producing supernatant (Fan lab, UC Irvine) [19], and an AlexaFluor 488 Goat-anti-Mouse secondary IgG (Life Technologies). Plates were scanned and colonies counted using the Fuji FLA-5100 scanner.

The viral genome plasmid pCMMP-luciferase was used to generate the Mo-MuLV reporter viruses and mZBS-Mo-MuLV reporter virus, as described previously [8]. Virus-containing supernatants were harvested 48 hr post-transfection and treated with DNase (40 U/ml) before aliquotting and freezing at −80°C.

### *In vivo* infections

Virus was harvested from extracellular supernatants from productively infected NIH3T3-43D cells and used to infect p2-p3 pups by intraperitonealinjection in a total volume of 25 μl Mo-MuLV (8×10^5^ IU/ml). For early time points, animals were sacrificed and spleen, thymus, and bone marrow were collected in order to quantify virus infection by FIA as described above. Briefly, single cell suspensions of each tissue were generated as described before and 10-fold serial dilutions of these cells were co-cultured with NIH3T3 cells until these cells reached confluency. Similar experiments were conducted with T-cells, B-cells, and T/B-depleted samples that were purified from pooled 10-day mouse bone marrow samples using the Dynabeads FlowComp Pan-T or Pan-B cell isolation kits (Life Technologies). The cells were then stained using antibodies described in [19].

### Tumor diagnosis and histopathological analysis

For tumor studies, 2–3 day old pups were infected as described above and monitored until they developed disease. Tumors were diagnosed as described previously [15]. Mice were observed daily for signs of lethargy, hunched posture, and scruffy fur associated with Mo-MuLV-induced tumors. Moribund animals were sacrificed and necropsy was performed. Spleen, thymus, liver, and kidney tissues were weighed and pieces of these organs along with lymph nodes were snap frozen and fixed in either 4% paraformaldehyde in PBS (US Biological) or 10% formaldehyde.

Fixed tissues were processed by Pacific Pathology, Inc. Fixed tissue samples were embedded in paraffin and 5 μm sections were cut and studied under light microscopy after hematoxylin-eosin (H&E) staining. Additional slides were prepared for immunofluorescence analysis by rehydrating slides, performing antigen retrieval using a sodium citrate buffer, and staining with primary rabbit antibodies to CD3 (Abcam) or CD79B (Santa Cruz Biotechnology) and with a secondary anti-rabbit antibody (Life Technologies).

## Abbreviations

ZASC1: Zinc finger amplified in esophageal squamous carcinoma 1; Mo-MuLV: Moloney murine leukemia virus; LK: Lin^-^Ska^-^Kit^+^; LSK: Lin^-^Ska^+^Kit^+^

## Competing interests

The authors declare that they have no competing interests.

## Authors’ contributions

SS, JB, ML, KFL, HF, PA, and JY conceived of the study and participated in its design. SS performed all experiments with the help of KL in making the mouse model and ML in diagnosing tumors. SS and JY wrote the manuscript and all authors read, edited and approved the final manuscript.

## Supplementary Material

Additional file 1: Figure S1 ZASC1 is not required for T-cell activation and proliferation. Mature CD4+ and CD8+ T-cells were isolated from mouse spleen and lymph nodes of 3-week-old ZASC1^+/+^ and ZASC1^−/−^ animals using α-CD4 and α-CD8 conjugated magnetic beads. Purified cells were labeled with CFSE (Carboxyfluorescein diacetate succinimidyl ester) and incubated with IL-2 on α-CD3, α-CD28 coated plates for 4 days to induce T-cell activation and proliferation. CFSE intensity decreases by 50% with each round of cell division leading to peaks representing different levels of proliferation. Flow cytometry histogram shows T-cell proliferation in a representative ZASC1^+/+^ (red) and ZASC1^−/−^ (blue) littermate control. Click here for file

Additional file 2: Figure S2 Flowchart of Hematopoietic Development. Hematopoietic stem cells (HSC) are the self-renewing cell type capable of repopulating the entire hematopoietic lineage. Cell surface molecules used to identify these cells are listed. Differentiation is a step-wise process in which cells lose repopulation potential and become functionally mature cells within the immune system. CMP, common myeloid progenitor; CLP, common lymphoid progenitor; MEP, megakaryocyte-erythroid progenitor; GMP, granulocyte-macrophage progenitor. Click here for file

Additional file 3: Figure S3 Virus titers (infectious centers/10^6^ cells) were measured from spleen, thymus, and bone marrow of Mo-MuLV infected animals collected at 7, 10 and 13 days post infection. Each dot represents one animal. ZASC1^+/+^ animals were not shown because the sample sizes were too small to draw meaningful conclusions. ZASC1^+/−^ (squares) and ZASC1^−/−^ (triangles). Mean average values indicated by horizontal bar. A.) Spleen B.) Thymus C.) Bone Marrow. Click here for file
