# Supplementary figures and images for: ZASC1 knockout mice exhibit an early bone marrow-specific defect in murine leukemia virus replication
Source: Virol J. 2013 Apr 24;10:130. doi: 10.1186/1743-422X-10-130 (PMC3654992; doi:10.1186/1743-422X-10-130)

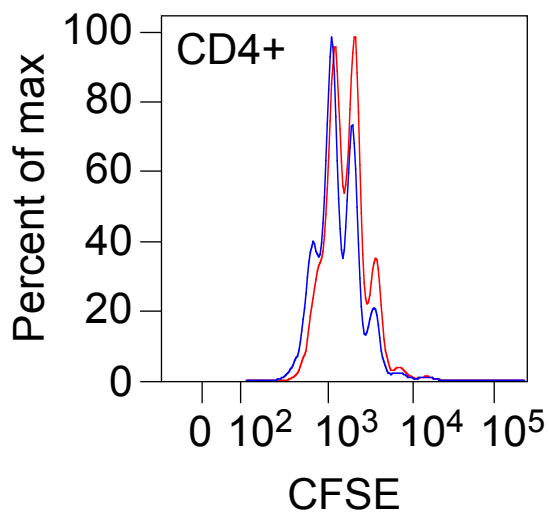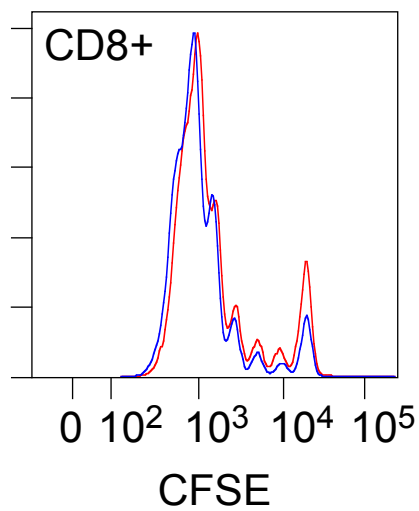

Supplement: Additional file 1: Figure S1 — ZASC1 is not required for T-cell activation and proliferation. Mature CD4+ and CD8+ T-cells were isolated from mouse spleen and lymph nodes of 3-week-old ZASC1+/+ and ZASC1−/− animals using α-CD4 and α-CD8 conjugated magnetic beads. Purified cells were labeled with CFSE (Carboxyfluorescein diacetate succinimidyl ester) and incubated with IL-2 on α-CD3, α-CD28 coated plates for 4 days to induce T-cell activation and proliferation. CFSE intensity decreases by 50% with each round of cell division leading to peaks representing different levels of proliferation. Flow cytometry histogram shows T-cell proliferation in a representative ZASC1+/+ (red) and ZASC1−/− (blue) littermate control. [file 1743-422X-10-130-S1.pdf]

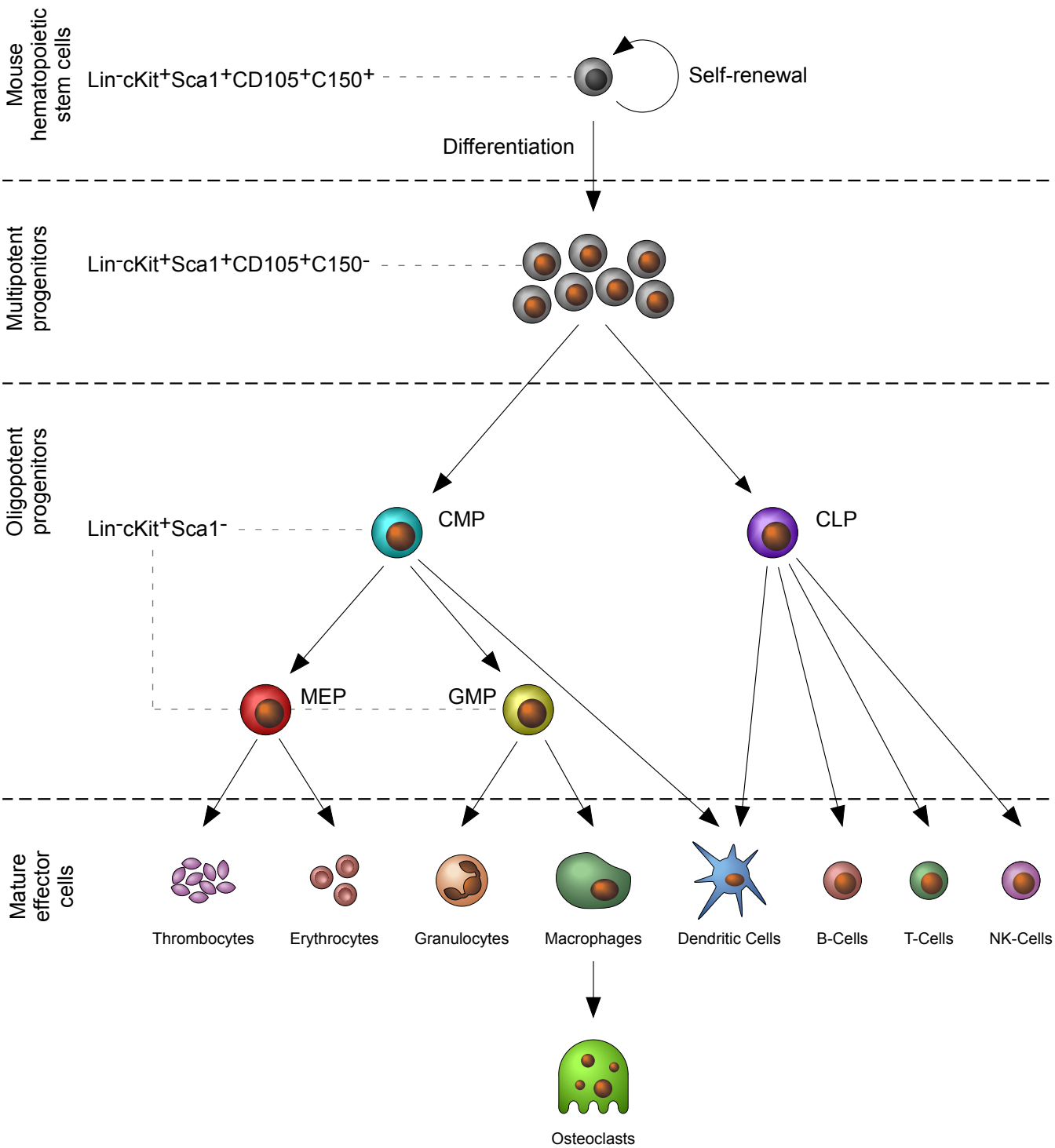

Supplement: Additional file 2: Figure S2 — Flowchart of Hematopoietic Development. Hematopoietic stem cells (HSC) are the self-renewing cell type capable of repopulating the entire hematopoietic lineage. Cell surface molecules used to identify these cells are listed. Differentiation is a step-wise process in which cells lose repopulation potential and become functionally mature cells within the immune system. CMP, common myeloid progenitor; CLP, common lymphoid progenitor; MEP, megakaryocyte-erythroid progenitor; GMP, granulocyte-macrophage progenitor. [file 1743-422X-10-130-S2.pdf]

**A**

7 day

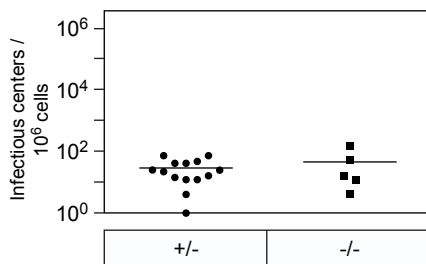

10 day

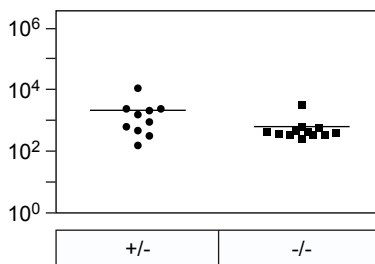

13 day

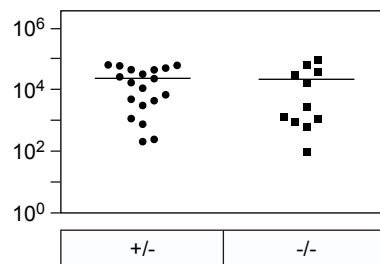**B**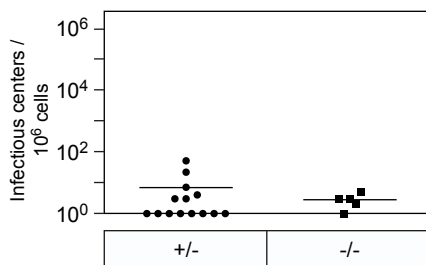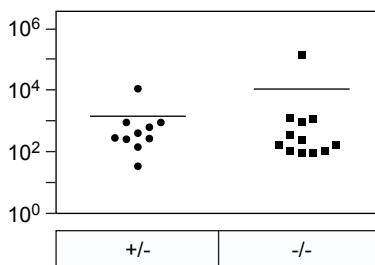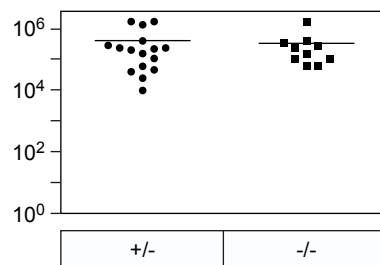**C**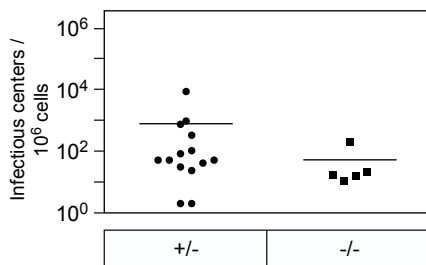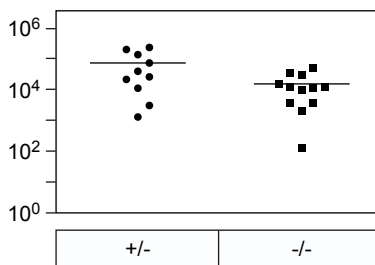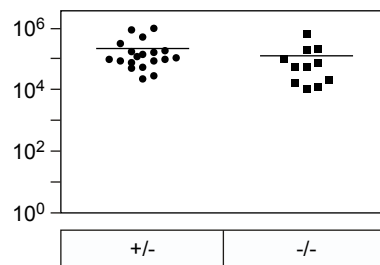

Supplement: Additional file 3: Figure S3 — Virus titers (infectious centers/106 cells) were measured from spleen, thymus, and bone marrow of Mo-MuLV infected animals collected at 7, 10 and 13 days post infection. Each dot represents one animal. ZASC1+/+ animals were not shown because the sample sizes were too small to draw meaningful conclusions. ZASC1+/− (squares) and ZASC1−/− (triangles). Mean average values indicated by horizontal bar. A.) Spleen B.) Thymus C.) Bone Marrow. [file 1743-422X-10-130-S3.pdf]
